# Measuring the capacity to combat illicit tobacco trade in 160 countries

**DOI:** 10.1186/s12992-021-00783-4

**Published:** 2021-11-17

**Authors:** Valerie Gilbert Ulep, Monica Paula Lavares, Ariza Francisco

**Affiliations:** 1 Philippine Institute for Development Studies, Quezon City, Philippines; 2 School of Government, Ateneo de Manila University, Quezon City, Philippines

**Keywords:** Tobacco, Illicit trade, Index, Smoking

## Abstract

**Background:**

Illicit trade of tobacco negatively affects countries’ tobacco control efforts. It leads to lower tobacco prices and makes tobacco products more accessible to vulnerable populations. In this study, we constructed an illicit tobacco trade index, which measures the structural and institutional capabilities of 160 countries in addressing illicit tobacco trade. We collected the most recent and best available data on general governance, tobacco control policies, and trade and customs practices.

**Results:**

Singapore, New Zealand, Finland and Sweden lead countries with the most favorable illicit tobacco trade score. We observed a positive relationship between illicit tobacco trade scores and Gross National Income (GNI) per capita and a negative relationship with the share of illicit tobacco trade to total tobacco consumption.

**Conclusions:**

The capability to combat illicit trade varies across countries. However, on average, low and middle-income countries (LMICs) are less capable of addressing illicit tobacco trade as suggested by the lower illicit tobacco trade index score. The lower index score in low and middle-income countries was mainly driven by low scores in tobacco control policies and trade and customs practices and conditions. Our study reinforces the importance for LMICs to adopt the WHO’s Protocol to Eliminate Illicit Tobacco Trade Products, particularly committing to treaty obligations and investing on track and trace system and other customs reforms.

**Supplementary Information:**

The online version contains supplementary material available at 10.1186/s12992-021-00783-4.

## Introduction

Tobacco smoking kills more than eight million people every year. Seven million of these preventable deaths are directly attributed to tobacco use and approximately 1.2 million to second-hand smoking [1]. Controlling illicit tobacco trade (ITT) together with progressive taxation and supply regulations are effective interventions to decrease the disease burden caused by smoking. ITT is defined as the supply, distribution, and sale of smuggled genuine or counterfeit tobacco products [2]. ITT negatively affects the tobacco control efforts of countries. It leads to lower tobacco prices and makes tobacco products; the availability of low-priced illicit tobacco products undermines tobacco control policy efforts intended to reduce the consumption of tobacco especially to young and poor populations [3]. Also, ITT causes significant damage government revenue from tobacco tax revenues [4–6]. Annually, governments lose an estimated US$40 billion in tax revenue from unreported illicit trade of tobacco products [7]. In some countries, illicit trade can be as high as 50 % of the overall tobacco market. It has been estimated that 1 in every 10 cigarettes and tobacco products consumed globally is illicit. ITT is more common in low and middle-income countries (LMICs) [7]. The estimated shares of illicit cigarettes to total cigarette consumption in low-income and middle-income countries were 16.8 % and 11.8 %, respectively compared to 9.8 % in high-income countries [8].

ITT could be attributed to multiple demand and supply factors. Demand factors refer to the preferences of smokers for cheaper or specific tobacco products, while supply factors refer to activities of legal and illegal business entities to increase profit, sales, and market shares enabled by the presence of corruption and organized crime, and weak government institutions [9]. Given the multi-dimensional construct of ITT, a holistic measure of these factors is needed in order to identify the appropriate policy response. We constructed an index, which measures the capacity of 160 economies in addressing ITT. The index score does not measure the effectiveness of countries in addressing illicit tobacco trade. Rather, the index examines their structural and institutional capacity to combat it. We have identified 29 indicators related to intellectual property rights, corruption, rule of law, organized crime, government effectiveness, informality, tax administration, tobacco tax policies, supply-side tobacco regulations, customs and trade practices. We further categorized these indicators into three: (1) general governance, (2) tobacco control policies, and (3) customs and trade practices.

There are several related indices such as illicit trade environment and tobacco interference indices [10, 11]. However, to our knowledge, the ITT index is the first of its kind that attempts to consider a wide-range of variables in the development of index specifically for illicit tobacco. The ITT Index could be used as a diagnostic tool for governments in examining countries’ capacity in addressing illicit tobacco trade. However, like most indices, it must be complemented with a more in-depth examination to fully understand the contextual challenges in addressing illicit trade of tobacco products in a particular country. It could also be used for benchmarking exercises amongst countries, which is effective in stimulating debate and call for action.

## Methods

**Fig. 1:**
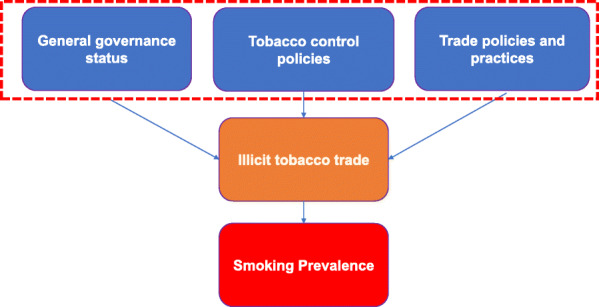
Theoretical Framework

We followed five steps in constructing an ITT index. The first step was developing a theoretical framework, which is essential in defining ITT and in identifying relationships of the different factors of illicit trade. Our framework informed the identification and selection of variables to be included in the index. We conducted an extensive literature search of quantitative studies to identify these different drivers of ITT. We did a search of PubMed using the search terms “Illicit tobacco trade”, “illegal cigarette”, “smuggling”, “factors”, “determinants”, “modelling”, in various combinations. We did not use any language or date restrictions. We did the same search on Google Scholar. We searched resources from relevant websites that publishes gray literature. We recorded all the factors that show positive relationships in quantitative studies. We categorized these factors into three domains: general governance, supply and demand tobacco control policies, and trade and customs practices. Figure 1 shows the theoretical framework we developed in this study, which demonstrates the relationships of different enabling factors, which lead to higher supply of illicit tobacco then higher tobacco consumption. General governance refers to structures and processes that are designed to ensure accountability, transparency, responsiveness, rule of law, stability, empowerment, and broad-based participation. Empirical studies have demonstrated that ineffective governance, corruption, instability of government, presence of organized crimes, weak law enforcement, presence of informal channels, and ineffective tax administration are determinants of ITT [9]. Typically, large scale illicit tobacco trade is conducted by organized criminal networks, flourishes in environments characterized by weak governance, high levels of corruption, and lax law enforcement [12, 13]. In environment with weak governance, transnational tobacco companies (TTC) complicity engage in large-scale cigarette smuggling as part of their strategy to increase profit [14]. Also, tobacco control policies such as imposition of higher taxes and supply-side interventions (that is, the adaption of smoke-free environment free regulations) also affects the demand for tobacco products. While the link between price and tax structure and ITT remains contentious, tobacco control policies are important factors affecting ITT. In general, ITT is higher in countries with lower cigarette prices and lower tax rates [15–19]. Lastly, customs and trade practices exacerbate the inherent challenges in addressing ITT [20]. Factors under this domain include the limited capacity of customs in terms of technology, tools and manpower to track trade and distribution. Weak customs governance, including corruption could facilitate smuggling and illegal trade of tobacco products [21–23].

The second step in constructing an index was variable selection. In general, the validity of indices is largely dependent on the quality of the underlying variables. In selecting relevant variables as measures of a particular construct (e.g., intellectual property), we only included data with well-documented and transparent methodology that can be accessed online. The methodology includes detailed data source and estimation procedures. We used latest survey estimates from the World Bank (e.g., World Enterprise Survey, World Logistics Survey), World Health Organization (e.g., tobacco prices and taxes estimates), and World Economic Forum (e.g., Global Competitiveness Survey). We have addressed missing data using group mean imputation [24]. By group mean imputation, we replaced missing data with the mean value of income grouping (that is, based GNI per capita) which the missing record belongs to. Appendix A material shows the variables we used in the construction of ITT index score, including the data source.

The third step was data normalization. We conducted data standardization using Min-Max method by subtracting the minimum value and dividing by the range of the indicator values. This is done by subtracting the minimum value of the indicator and dividing by the range of the indicator values:

$${I}_{ij}=\frac{{x}_{ij}-\text{min}\left({x}_{i}\right)}{\text{max}\left({x}_{i}\right)-\text{min}\left({x}_{i}\right)}$$

where ${I}_{ij}$ is the standardized value of the $i$th indicator for the $j$th country

${x}_{ij}$ is the actual value of the $i$th indicator for the $j$th country

$min\left({x}_{i}\right)$ is the minimum value of the $i$th indicator across all countries

$max\left({x}_{i}\right)$ is the maximum value of the $i$th indicator across all countries

This method transformed the indicators previously measured in different scales into normalized indices with identical range of values between 0 and 1 [20].

The fourth step was data aggregation. Indicators under each domain were then aggregated to obtain a single measurement score per dimension. Under the normative assumption that each domain is equally important in the assessment as a whole, equal weighting is applied in aggregating the individual indicators under each domain. Hence, the maximum score in each dimension is 33.3 % or, more accurately, 1/3. In equal weighting, each domain has the same importance when determining the index’s value. Typically, composite indices rely on equal weighting largely because of the insufficient empirical or quantitative basis of the weights. In this study, while unequal weighting seems appropriate given the contextual differences across countries, the lack of consensus on weights makes unequal weighting seems justifiable.

The overall score was computed as the geometric mean of the three-dimension indices. We used geometric mean because it offers a partial compensability between indicators. The geometric aggregation formula of sub-indicators to compute the composite score (ITT) takes the following form:

$$\text{I}\text{T}\text{T}=exp\frac{\sum _{j}^{n}{w}_{j}\text{l}\text{n}\left({SI}_{j}\right)}{{\sum }_{j=1}^{n}{w}_{j}}$$

ITT is the composite index to be computed, ${w}_{j}$is the relative weight of the sub-domain ${SI}_{j}$, n is the number of sub-domain (*n*=3) aggregated to form the composite indicator and exp and ln are the exponential and logarithmic functions respectively. This approach in creating a composite score using equal weighting and geometric aggregation is similar to the methodology used by the Human Development Index (HDI). The higher the ITT score (that is, 0=lowest; 1=highest) the less vulnerable to illicit tobacco trade. There are various aggregation methods discussed elsewhere including their advantages and disadvantages [24].

Lastly, we conducted bivariate analysis with the share of illicit tobacco trade to tobacco consumption. We obtained data on illicit tobacco trade and smoking prevalence from Euromonitor International and World Health Organization, respectively. Despite its potential limitations, we used data on illicit tobacco trade from Euromonitor International in order check the validity of ITT because it is the only source of complete and comparable data on value and volume on illicit trade across a large number of countries.

While the index captures the enabling factors of ITT, it is important to triangulate the ITT Index scores with the size of the tobacco market in a particular country. Here, we adjusted the ITT score with smoking prevalence of the country using the formula *[adjusted ITT=(1-(prevalence/100)) x ITT overall score*], which should yield to a more realistic score. By applying this formula, countries with high prevalence of smoking will lead to lower adjusted ITT score, meaning more vulnerable to ITT.

## Results

Table 1 shows the mean values of variables included in constructing ITT scores for 160 countries disaggregated by income group (i.e., Gross National Income per capita). The countries were categorized as low-income if GNI per capita is $1,025 or less in 2018; lower middle-income economies are those with a GNI per capita between $1,026 and $3,995; upper middle-income economies are those between $3,996 and $12,375; high-income economies are those with a GNI per capita of $12,376 or more. Our descriptive analysis suggests that GNI per capita is highly correlated with trade and custom practices and governance, tobacco control policies, and general governance indicators. Relative to other low and income countries, high-income countries have relatively favorable trade and customs practices and general governance environments. Also, they have better tobacco control policies using demand and supply policy interventions, including higher tobacco prices, higher taxes, and lower tobacco price dispersion. Lastly, they are more compliant to smoke-free environment policies.

**Table 1:** Variables used for the construction of ITTScore index

Domains	Sub-domain	Variable	OECD	Non-OECD	LIC	LMIC	UMIC	World
Governance	Intellectual property	Extent of intellectual property rights (lowest: 1; highest: 7)	5.4	4.8	3.5	3.7	3.8	4.2
		Extent are property rights, including financial assets, protected (lowest: 1; highest: 7)	5.4	4.9	3.7	3.9	4.0	4.3
	Corruption	Firms experiencing bribes (%)	2.9	5.7	23.6	23.5	12.6	14.3
		Public transactions where a gift or informal payment was requested (%)	2.1	4.2	18.4	19.0	9.8	11.2
		Firms expected to give gifts in meetings with tax officials (%)	1.0	3.7	18.6	17.9	10.3	10.9
		Firms expected to give gifts to secure government contract (%)	7.0	3.5	36.7	31.8	20.1	21.0
		Firms expected to give gifts to get an operating license (%)	3.1	5.9	20.8	18.3	9.7	11.8
		% of firms expected to give gifts to get an import license	0.4	4.0	17.4	19.2	9.6	10.7
		% of firms expected to give gifts to public officials "to get things done"	7.9	9.6	30.2	32.7	13.1	19.2
		% of firms identifying the courts system as a major constraint	12.0	14.3	39.9	39.9	34.4	29.8
	Rule of law	Favoritism of government officials to well-connected firms and individuals when deciding upon policies and contracts (lowest: 1; highest: 7)	3.9	3.7	2.9	3.0	2.8	3.2
		Independence of judicial system from influences of the government, individuals, or companies (lowest: 1; highest: 7)	5.3	4.6	3.2	3.5	3.5	4.0
	Organized crime	Extent of organized crime (mafia-oriented racketeering, extortion) impose costs on businesses (lowest: 1; highest: 7)	5.6	5.7	4.3	4.3	4.3	4.7
		Reliability of police services (lowest: 1; highest: 7)	5.7	5.2	3.8	3.8	3.9	4.4
	Government effectiveness	Extent of public trust in politicians (lowest: 1; highest: 7)	4.0	3.9	2.8	2.9	2.6	3.1
		Extent of burden of government regulation (lowest: 1; highest: 7)	3.5	3.9	3.6	3.4	3.2	3.5
		Transparency of government policymaking (lowest: 1; highest: 7)	4.9	4.7	3.7	3.8	4.0	4.2
	Informality	Firms competing against unregistered or informal firms (%)	28.6	44.9	61.5	55.4	50.6	48.5
		Firms identifying practices of competitors in the informal sector as a major constraint (%)	16.2	21.0	35.4	32.1	28.1	27.0
	Tax administration	Percent of firms identifying tax administration as a major constraint (%)	18.5	14.0	27.8	25.6	23.0	22.4
Tobacco control policies	Demand-related	Price of cigarette per pack (in USD)	8.7	8.9	3.3	3.9	6.6	6.1
		Tax burden of cigarette (% share of retail price)	56.4	40.9	20.1	33.1	40.8	38.7
		Affordability (% share of per capita income)	2.7	2.0	18.0	7.6	4.3	6.8
		Price dispersion Share of cheapest brand price in premium brand price (%) (the higher the % the smaller the gap)	77.1	59.9	28.4	42.6	53.5	52.2
	Supply restriction	Compliance to smoke free environment (lowest: 0; highest: 10)	8.5	8.5	3.8	4.2	5.6	5.9
Trade policies and customs practices	Trade policies and customs practices	Efficiency of customs and border management clearance (lowest: 1; highest: 5)	3.5	3.0	2.2	2.4	2.5	2.7
		Quality of trade and transport infrastructure (lowest: 1; highest: 5)	3.7	3.1	2.1	2.3	2.6	2.7
		Competence and quality of logistics services (lowest: 1; highest: 5)	3.7	3.1	2.3	2.5	2.6	2.8
		Ability to track and trace consignments (lowest: 1; highest: 5)	3.8	3.1	2.4	2.6	2.7	2.9

Note: OECD= Organization for Economic Co-operation and Development; LIC=Low Income Country; LMIC=Lower Middle-Income Country; UPMIC=Upper Middle-Income Country; HIC=High Income Country

**Fig. 2:**
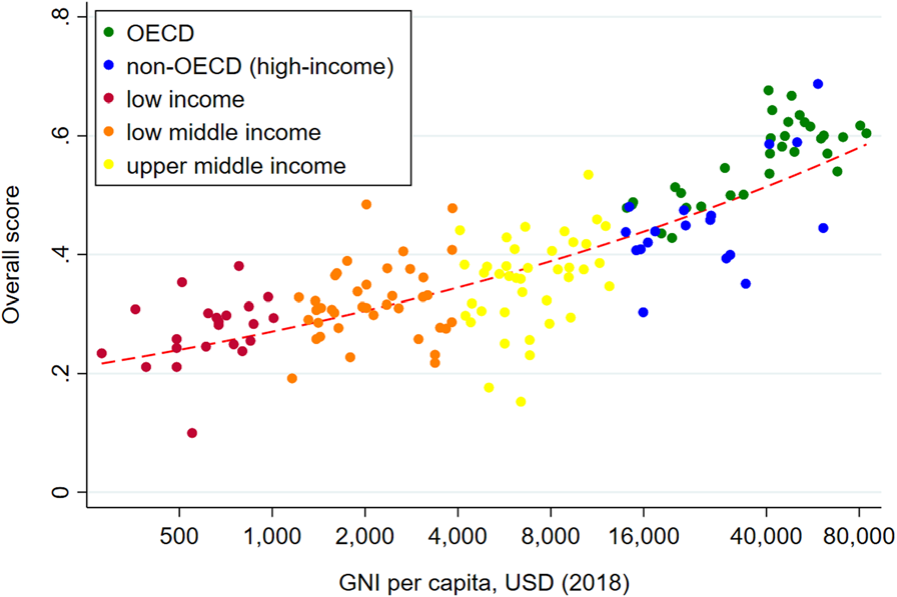
ITT Index Score vs. Share of Illicit Tobacco Trade

ITT Index is ranked on a scale from 0 to 1.0, with 1.0 being the highest capacity score to address illicit tobacco. Table 2 shows the ITT Index scores by domain and income group. On average, high-income countries tend to have higher overall ITT scores compared to LMICs (median overall score: 0.55 vs. 0.31). Figure 2 shows the negative relationship between GNI per capita and overall score (*R*=67; *p*=0.000). While high-income countries tend to have higher scores across the three domains (i.e., general governance, tobacco control policies, and trade and customs practices), their contribution to the overall score markedly vary across income group. For instance, trade and customs practices appear to be the major driver of higher scores in high-income countries, but this is not the case for LMICs. Trade and customs practices and tobacco control policies appear to be the major drivers of relatively low ITT scores in low-income countries (median: 0.22). In Appendix B shows the overall and domain ITT Index score for 160 countries.

**Table 2:** ITT index, by income group

	General governance	Tobacco control policies	Trade and customs	Overall (adjusted)
High-income: OECD	0.45	0.54	0.79	0.57
High-income: non-OECD	0.40	0.47	0.49	0.44
Low-income	0.38	0.26	0.22	0.28
Lower middle-income	0.38	0.28	0.30	0.31
Upper middle-income	0.34	0.40	0.36	0.36
**World**	**0.38**	**0.39**	**0.37**	**0.36**

Table 3 shows the countries with the highest ITT index score ranked by country disaggregated by income group. The countries with the most favorable ITT scores are the following: Singapore, New Zealand, Australia, Finland and Sweden. While countries with least favorable ITT Index scores are Afghanistan, Libya, Iraq.

**Table 3:** Top countries with highest ITT index

Overall rank	Country	GNI per capita (2018) in USD	General governance	Tobacco control policies	Trade and customs	Overall (adjusted)
High-income countries (OECD and non-OECD)						
1	Singapore	58,770	0.90	0.66	0.91	0.69
2	New Zealand	40,640	0.90	0.69	0.86	0.69
3	Australia	53,230	0.82	0.65	0.83	0.67
4	Finland	48,280	0.91	0.62	0.90	0.66
5	Sweden	55,490	0.87	0.52	0.91	0.65
Low and middle-income countries (LMICs)						
54	India	2,020	0.61	0.43	0.54	0.49
53	Sri Lanka	4,060	0.63	0.66	0.35	0.47
41	Malaysia	10,590	0.66	0.46	0.56	0.45
49	South Africa	5,750	0.73	0.34	0.60	0.44
47	Mauritius	12,050	0.70	0.51	0.45	0.44

We examined the relationship between the share ITT and ITT Index score countries with 2017 data from Euromonitor International. Of the 160 countries included in the analysis, we only have data on illicit trade for 78 countries. The average share of illicit trade is 17 % ranging from 4 to 54 %. Our bivariate analysis shows a statistically significant correlation between ITT score and the share of ITT to total consumption (*R*=0.56; p value:0.03). The average ITT score for those countries with higher share of ITT (that is, above the global mean: 16 %) is 0.35 compared to 0.48 in countries with lower shares. However, the caveats of available ITT estimates should be taken with caution. There is almost always a large discrepancy across estimates from academia, tobacco industry, and other organizations. Based on empirical studies, industry estimates of illicit tobacco is likely higher than those from academic studies. For example, Clarke and Prentice (2012) noted that the discrepancy between estimates from the industry-commissioned studies relative to academic studies (16 % versus 3 %) [25].

**Fig. 3:**
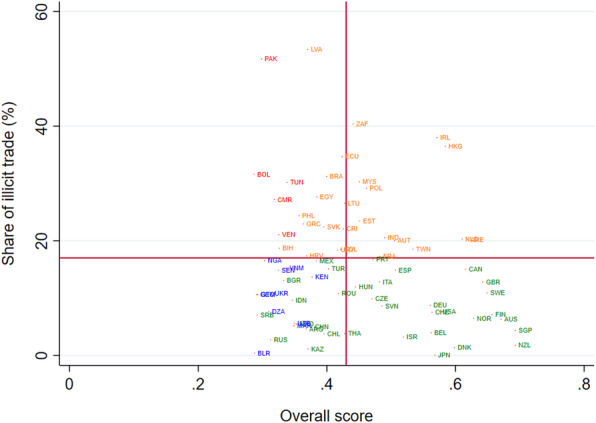
ITT Index Score and Share of Illicit Trade

Figure 3 shows the statistically significant relationship between adjusted ITT Index score and ITT as share of total cigarette consumption (p value: 0.04). The ITT index scores of European countries especially those with high smoking prevalence, particularly Greece, Estonia, Croatia, Cyprus, and Czech Republic have large decline in their ITT scores. For middle-income countries, we observed substantially lower adjusted ITT score for Indonesia and Turkey, both countries with relatively high smoking prevalence. After adjustment, the mean overall ITT score declined from 0.38 to 0.31 across 160 countries. The ITT score of high-income OECD countries decreased from 0.55 to 0.43. While LMICs have small difference in adjusted and adjusted ITT index scores. See Appendix B for the adjusted and unadjusted ITT Index Score with the domain score of general governance, tobacco control policies, and trade and customs practices for each 160 countries.

## Discussion

To measure the overall capacity of countries in addressing illicit tobacco, we constructed the Illicit Tobacco Trade (ITT) Index, which is the average capacity of countries in three dimensions: (1) general governance, (2) tobacco control policies, and (3) trade and customs practices. The ITT Index score is the geometric mean of normalized indices of the three dimensions.

ITT index is ranked on a scale from 0 to 1.0, with 1.0 being the highest capacity score to address illicit tobacco. The average global ITT Index score is 0.36, with a large variation across countries. The relatively low global average suggests that many countries around the world have a significant room to improve their capacity in addressing illicit tobacco trade. On average, low and middle-income countries (LMICs) have lower capacity compared to their high-income counterparts in addressing ITT. Our bivariate analysis shows a strong positive relationship between ITT scores and GNI per capita. However, it is interesting to note that there are several LIC and LMIC that have impressively high ITT Index scores. A deep-dive analysis could be performed to understand why these countries have higher ITT Index score compared to other countries with more or less similar income.

The relatively higher vulnerability of LMICs could have contributed to the proliferation of illicit tobacco products in developing countries, which made tobacco more accessible and affordable especially to vulnerable populations. The tobacco epidemic is growing in developing countries largely because of weak tobacco control policies and the globalization of the tobacco industry. In recent decades, the growing presence of transnational tobacco corporations (TTC) in developing countries has changed the production and marketing of tobacco products in emerging markets. Reports suggest that TTCs have contributed to the increasing demand for tobacco by undermining regulations, and in some jurisdictions, and have engaged in illicit trade such as smuggling to maximize profit [14].

The ITT Index score is positively correlated with national income. However, the contribution of different dimensions to the overall ITT Index scores varies across income groups. For example, in HIC, the trade and custom domain appears to be the driver of a higher ITT Index score. In contrast, tobacco control policies and trade and customs practices domains are drivers of LMICs and UMICs. The lower index score in tobacco control domain among low and middle-income countries reinforces the need to implement the WHO Framework for Tobacco Control (FCTC), particularly the adoption of high and simple tobacco taxes. On average, prices of tobacco remain affordable and tax burden are substantially lower in developing countries [26]. Political will is needed in legislating progressive tobacco tax regime. However, governments are more often hesitant in imposing higher taxes out of fear of possible increase in higher illicit tobacco activities, a scare tactic commonly used by the tobacco industry to block tax reforms [27]. While the lower scores on customs and trade highlights the importance for developing countries to adopt the WHO’s Protocol to Eliminate Illicit Tobacco Trade Products. For example, developing countries should commit to the treaty obligations and invest on systems such as track and trace and other customs reforms.

The ITT Index score does not measure the magnitude of illicit tobacco, but could be used to diagnose the capacity of countries to address the problem. It should stimulate discussions among policy makers and researchers of countries, and consider it as a diagnostic tool and precursor in conducting a more in-depth analysis of the different adaptive challenges of addressing illicit tobacco. This should be complemented with mixed method designs to fully understand the drivers of illicit tobacco trade in a given jurisdictions. For example, in the case of Afghanistan and Pakistan, two countries considering the relatively lower tax regime and complex political systems and criminal actors in the former, which exacerbates tobacco smuggling [28].

Our study has the following limitations. First, we conducted imputation of missing data for selected variables because of the lack available data. While our imputation method is a commonly used approach, no imputation model is free of assumption and possible bias. Mean group imputations typically reduces variance and could distort relationships between variables.

In our study, the bivariate analysis of ITT score and share of illicit tobacco trade could have resulted into a bias estimate. Second, like most indices, there are innate limitations of different aggregation approaches. We used geometric approach for aggregation with the attempt to address the compensatory problem commonly found in other methods such as linear aggregation methods. Typically, countries with low scores in a particular sub-domain would prefer a linear approach rather than geometric as the domain with high score will compensate the domain with low score. The geometric method reduces this problem; we assumed that all domains are conceptually equal. However, based on empirical studies, some variables have more economical, clinical and social relevance over others. Further studies are needed to address this possible methodological limitation.

## Conclusions

We constructed an index, which aims to guide governments in measuring their overall capability in addressing illicit tobacco trade. Our findings suggest the highly heterogenous capacity across countries. LMICs, on average, appear to be less capable in addressing illicit tobacco trade as suggested by the relatively low scores. The relatively lower index score in LMICs was largely driven by low scores in tobacco control policies and trade and customs practices and conditions. This therefore reinforces the need to fully implement the FCTC Protocol WHO’s Protocol to Eliminate Illicit Tobacco Trade Products in LMICs particularly the adoption of large and simple tax rates for tobacco and customs reforms.

## Supplementary information

**Additional file 1.**

## Data Availability

The datasets generated and/or analyzed during the current study are available in the: World Economic Forum’s Global Competitiveness Survey 2017-2018: http://reports.weforum.org/global-competitiveness-index-2017-2018/competitiveness-rankings/. World Bank’s World Enterprise Survey: https://www.enterprisesurveys.org/en/enterprisesurveys. World Bank’s World Bank Logistics Survey: https://lpi.worldbank.org/. World Health Organization: WHO report on the global tobacco epidemic 2019. https://www.who.int/teams/health-promotion/tobacco-control/who-report-on-the-global-tobacco-epidemic-2019.
