# Supplementary material for: Measuring the capacity to combat illicit tobacco trade in 160 countries
Source: Global Health. 2021 Nov 17;17:130. doi: 10.1186/s12992-021-00783-4 (PMC8597249; doi:10.1186/s12992-021-00783-4)
Supplement: Supplementary file 1 — Additional file 1. [file 12992_2021_783_MOESM1_ESM.docx]

**Appendix A. Variables used for the ITT Index score**

| **Domains** | **Sub-domain** | **Variable** | **Source** |
| --- | --- | --- | --- |
| Governance | Intellectual property | Extent of intellectual property rights (lowest: 1; highest: 7) | World Economic Forum’s Global Competitiveness Survey 2017-2018 (World Economic Forum, 2020) |
|  |  | Extent are property rights, including financial assets, protected (lowest: 1; highest: 7) |  |
|  | Corruption | Firms experiencing bribes (%) | Analysis of World Bank’s World Enterprise Survey (various years) (World Bank, 2020) |
|  |  | Public transactions where a gift or informal payment was requested (%) |  |
|  |  | Firms expected to give gifts in meetings with tax officials (%) |  |
|  |  | Firms expected to give gifts to secure government contract (%) |  |
|  |  | Firms expected to give gifts to get an operating license (%) |  |
|  |  | % of firms expected to give gifts to get an import license |  |
|  |  | % of firms expected to give gifts to public officials "to get things done" |  |
|  |  | % of firms identifying the courts system as a major constraint |  |
|  | Rule of law | Favoritism of government officials to well-connected firms and individuals when deciding upon policies and contracts (lowest: 1; highest: 7) | World Economic Forum’s Global Competitiveness Survey 2017-2018 (World Economic Forum, 2020) |
|  |  | Independence of judicial system from influences of the government, individuals, or companies (lowest: 1; highest: 7) |  |
|  | Organized crime | Extent of organized crime (mafia-oriented racketeering, extortion) impose costs on businesses (lowest: 1; highest: 7) | World Economic Forum’s Global Competitiveness Survey 2017-2018 (World Economic Forum, 2020) |
|  |  | Reliability of police services (lowest: 1; highest: 7) |  |
|  | Government effectiveness | Extent of public trust in politicians (lowest: 1; highest: 7) | World Economic Forum’s Global Competitiveness Survey 2017-2018 (World Economic Forum, 2020) |
|  |  | Extent of burden of government regulation (lowest: 1; highest: 7) |  |
|  |  | Transparency of government policymaking (lowest: 1; highest: 7) |  |
|  | Informality | Firms competing against unregistered or informal firms (%) | World Economic Forum’s Global Competitiveness Survey 2017-2018 (World Economic Forum, 2020) |
|  |  | Firms identifying practices of competitors in the informal sector as a major constraint (%) |  |
|  | Tax administration | Percent of firms identifying tax administration as a major constraint (%) | Analysis of World Bank’s World Enterprise Survey (various years) (World Bank, 2020) |
| Tobacco control policies | Demand-related | Price of cigarette per pack (in USD) | World Health Organization (2018) |
|  |  | Tax burden of cigarette (% share of retail price) |  |
|  |  | Affordability (% share of per capita income) |  |
|  |  | Price dispersion Share of cheapest brand price in premium brand price (%) (the higher the % the smaller the gap) |  |
|  | Supply restriction | Compliance to smoke free environment (lowest: 0; highest: 10) |  |
| Trade policies and customs practices | Trade policies and customs practices | Efficiency of customs and border management clearance (lowest: 1; highest: 5) | World Bank Logistics Performance Survey (2018)  (Arvis, Ojali, Wiederer, Raj, & Dairabayeva, 2018) |
|  |  | Quality of trade and transport infrastructure (lowest: 1; highest: 5) |  |
|  |  | Competence and quality of logistics services (lowest: 1; highest: 5) |  |
|  |  | Ability to track and trace consignments (lowest: 1; highest: 5) |  |

**Appendix B. ITT Index score, by country**

| Income | Region | Gross National Product per capita | General Governance Index score | Tobacco control policies Index score | Trade and customs Index score | ITT Index score (un-adjusted) | ITT index score (adjusted) |
| --- | --- | --- | --- | --- | --- | --- | --- |
| Singapore | High income: non-OECD | 58770 | 0.90 | 0.66 | 0.91 | 0.81 | 0.69 |
| New Zealand | High income: OECD | 40640 | 0.90 | 0.69 | 0.86 | 0.81 | 0.69 |
| Australia | High income: OECD | 53230 | 0.82 | 0.65 | 0.83 | 0.76 | 0.67 |
| Finland | High income: OECD | 48280 | 0.91 | 0.62 | 0.90 | 0.80 | 0.66 |
| Sweden | High income: OECD | 55490 | 0.87 | 0.52 | 0.91 | 0.75 | 0.65 |
| United Kingdom | High income: OECD | 41770 | 0.86 | 0.62 | 0.89 | 0.78 | 0.64 |
| Norway | High income: OECD | 80610 | 0.89 | 0.59 | 0.78 | 0.74 | 0.63 |
| United Arab Emirates | High income: non-OECD | 40880 | 0.87 | 0.46 | 0.85 | 0.70 | 0.62 |
| Canada | High income: OECD | 44940 | 0.83 | 0.53 | 0.81 | 0.71 | 0.62 |
| Netherlands | High income: OECD | 51260 | 0.87 | 0.56 | 0.92 | 0.77 | 0.61 |
| Denmark | High income: OECD | 60140 | 0.83 | 0.51 | 0.90 | 0.73 | 0.60 |
| Hong Kong SAR, China | High income: non-OECD | 50300 | 0.86 | 0.47 | 0.86 | 0.70 | 0.58 |
| Iceland | High income: OECD | 67960 | 0.84 | 0.56 | 0.60 | 0.66 | 0.58 |
| Luxembourg | High income: OECD | 70870 | 0.92 | 0.54 | 0.76 | 0.72 | 0.58 |
| United States | High income: OECD | 63080 | 0.81 | 0.48 | 0.87 | 0.70 | 0.58 |
| Ireland | High income: OECD | 61390 | 0.85 | 0.65 | 0.69 | 0.73 | 0.57 |
| Japan | High income: OECD | 41310 | 0.84 | 0.49 | 0.93 | 0.72 | 0.57 |
| Switzerland | High income: OECD | 84410 | 0.90 | 0.49 | 0.87 | 0.73 | 0.56 |
| Belgium | High income: OECD | 45910 | 0.82 | 0.55 | 0.88 | 0.74 | 0.56 |
| Germany | High income: OECD | 47090 | 0.82 | 0.54 | 0.99 | 0.76 | 0.56 |
| Panama | High income: non-OECD | 14370 | 0.66 | 0.50 | 0.58 | 0.58 | 0.55 |
| Taiwan, China | High income: non-OECD | . | 0.76 | 0.47 | 0.74 | 0.64 | 0.53 |
| Saudi Arabia | High income: non-OECD | 21600 | 0.78 | 0.50 | 0.49 | 0.58 | 0.52 |
| Israel | High income: OECD | 40920 | 0.81 | 0.55 | 0.66 | 0.67 | 0.52 |
| Spain | High income: OECD | 29340 | 0.71 | 0.56 | 0.81 | 0.69 | 0.51 |
| Austria | High income: OECD | 49310 | 0.82 | 0.47 | 0.90 | 0.70 | 0.51 |
| India | Lower middle income | 2020 | 0.61 | 0.43 | 0.54 | 0.52 | 0.49 |
| Slovenia | High income: OECD | 24580 | 0.71 | 0.54 | 0.61 | 0.62 | 0.49 |
| France | High income: OECD | 41080 | 0.78 | 0.54 | 0.84 | 0.70 | 0.48 |
| Italy | High income: OECD | 33730 | 0.59 | 0.53 | 0.78 | 0.63 | 0.48 |
| Qatar | High income: non-OECD | 61150 | 0.86 | 0.27 | 0.64 | 0.53 | 0.48 |
| Portugal | High income: OECD | 21990 | 0.70 | 0.51 | 0.69 | 0.63 | 0.47 |
| Czech Republic | High income: OECD | 20240 | 0.70 | 0.53 | 0.72 | 0.65 | 0.47 |
| Bahrain | High income: non-OECD | 21890 | 0.79 | 0.46 | 0.44 | 0.54 | 0.47 |
| Sri Lanka | Upper middle income | 4060 | 0.63 | 0.66 | 0.35 | 0.52 | 0.47 |
| Oman | High income: non-OECD | 15140 | 0.77 | 0.30 | 0.52 | 0.49 | 0.47 |
| Poland | High income: OECD | 14100 | 0.63 | 0.56 | 0.66 | 0.61 | 0.46 |
| Estonia | High income: OECD | 21140 | 0.83 | 0.50 | 0.59 | 0.63 | 0.45 |
| Malaysia | Upper middle income | 10590 | 0.66 | 0.46 | 0.56 | 0.56 | 0.45 |
| Hungary | High income: OECD | 14780 | 0.64 | 0.58 | 0.66 | 0.63 | 0.44 |
| Malta | High income: non-OECD | 26480 | 0.74 | 0.59 | 0.43 | 0.57 | 0.44 |
| South Africa | Upper middle income | 5750 | 0.73 | 0.34 | 0.60 | 0.53 | 0.44 |
| Mauritius | Upper middle income | 12050 | 0.70 | 0.51 | 0.45 | 0.54 | 0.44 |
| Thailand | Upper middle income | 6610 | 0.65 | 0.37 | 0.62 | 0.53 | 0.43 |
| Lithuania | High income: non-OECD | 17430 | 0.71 | 0.55 | 0.48 | 0.57 | 0.43 |
| Costa Rica | Upper middle income | 11520 | 0.65 | 0.40 | 0.40 | 0.47 | 0.43 |
| Bahamas, The | High income: non-OECD | 30520 | 0.69 | 0.47 | 0.31 | 0.46 | 0.43 |
| Brunei Darussalam | High income: non-OECD | 29660 | 0.70 | 0.44 | 0.37 | 0.49 | 0.42 |
| Ecuador | Upper middle income | 6110 | 0.52 | 0.62 | 0.45 | 0.52 | 0.42 |
| Rwanda | Low income | 780 | 0.89 | 0.25 | 0.42 | 0.46 | 0.42 |
| Colombia | Upper middle income | 6180 | 0.51 | 0.42 | 0.44 | 0.45 | 0.42 |
| Romania | Upper middle income | 11290 | 0.59 | 0.56 | 0.49 | 0.55 | 0.42 |
| Uruguay | High income: non-OECD | 15650 | 0.71 | 0.55 | 0.36 | 0.52 | 0.42 |
| Cyprus | High income: non-OECD | 26300 | 0.69 | 0.54 | 0.52 | 0.58 | 0.40 |
| Turkey | Upper middle income | 10420 | 0.65 | 0.50 | 0.53 | 0.56 | 0.40 |
| Brazil | Upper middle income | 9140 | 0.46 | 0.44 | 0.47 | 0.46 | 0.40 |
| Jamaica | Upper middle income | 4970 | 0.52 | 0.54 | 0.30 | 0.44 | 0.40 |
| Chile | High income: OECD | 14670 | 0.74 | 0.51 | 0.59 | 0.61 | 0.40 |
| Slovak Republic | High income: OECD | 18260 | 0.61 | 0.52 | 0.50 | 0.54 | 0.40 |
| Jordan | Upper middle income | 4200 | 0.72 | 0.41 | 0.37 | 0.48 | 0.39 |
| Egypt, Arab Rep. | Lower middle income | 2800 | 0.64 | 0.39 | 0.41 | 0.47 | 0.38 |
| Mexico | Upper middle income | 9180 | 0.50 | 0.36 | 0.48 | 0.44 | 0.38 |
| Kenya | Lower middle income | 1620 | 0.55 | 0.30 | 0.42 | 0.41 | 0.38 |
| China | Upper middle income | 9460 | 0.70 | 0.23 | 0.73 | 0.49 | 0.38 |
| Kuwait | High income: non-OECD | 34290 | 0.68 | 0.28 | 0.43 | 0.44 | 0.37 |
| Eritrea | Low income | . | 0.70 | 0.52 | 0.16 | 0.39 | 0.37 |
| Kazakhstan | Upper middle income | 8070 | 0.67 | 0.45 | 0.37 | 0.48 | 0.37 |
| Latvia | High income: non-OECD | 16510 | 0.67 | 0.57 | 0.44 | 0.55 | 0.37 |
| Croatia | High income: non-OECD | 14000 | 0.62 | 0.51 | 0.52 | 0.55 | 0.37 |
| Argentina | Upper middle income | 12390 | 0.50 | 0.46 | 0.42 | 0.46 | 0.37 |
| Korea, Rep. | High income: OECD | 30600 | 0.69 | 0.49 | 0.75 | 0.63 | 0.36 |
| Turkmenistan | Upper middle income | 6740 | 0.58 | 0.59 | 0.27 | 0.45 | 0.36 |
| Iran, Islamic Rep. | Upper middle income | . | 0.59 | 0.26 | 0.42 | 0.40 | 0.36 |
| Greece | High income: OECD | 19770 | 0.59 | 0.58 | 0.53 | 0.57 | 0.36 |
| Côte d'Ivoire | Lower middle income | 1600 | 0.48 | 0.26 | 0.52 | 0.40 | 0.36 |
| Peru | Upper middle income | 6470 | 0.47 | 0.44 | 0.30 | 0.40 | 0.36 |
| São Tomé and Principe | Lower middle income | 1890 | 0.53 | 0.27 | 0.37 | 0.37 | 0.36 |
| Dominican Republic | Upper middle income | 7760 | 0.49 | 0.35 | 0.34 | 0.39 | 0.36 |
| Philippines | Lower middle income | 3830 | 0.55 | 0.42 | 0.42 | 0.46 | 0.36 |
| Montenegro | Upper middle income | 8430 | 0.58 | 0.39 | 0.36 | 0.44 | 0.35 |
| Macedonia, FYR | Upper middle income | 5450 | 0.61 | 0.39 | 0.35 | 0.44 | 0.35 |
| Togo | Low income | 660 | 0.57 | 0.37 | 0.25 | 0.37 | 0.35 |
| Fiji | Upper middle income | 5860 | 0.63 | 0.54 | 0.26 | 0.45 | 0.35 |
| Uzbekistan | Lower middle income | 2020 | 0.67 | 0.28 | 0.32 | 0.39 | 0.35 |
| Morocco | Lower middle income | 3090 | 0.59 | 0.36 | 0.30 | 0.40 | 0.35 |
| Bhutan | Lower middle income | 3080 | 0.81 | 0.42 | 0.20 | 0.41 | 0.35 |
| Indonesia | Lower middle income | 3840 | 0.59 | 0.42 | 0.51 | 0.50 | 0.35 |
| Honduras | Lower middle income | 2350 | 0.50 | 0.41 | 0.33 | 0.41 | 0.35 |
| El Salvador | Lower middle income | 3820 | 0.51 | 0.36 | 0.28 | 0.37 | 0.34 |
| Vietnam | Lower middle income | 2360 | 0.54 | 0.19 | 0.59 | 0.40 | 0.34 |
| Djibouti | Lower middle income | 3190 | 0.61 | 0.30 | 0.34 | 0.39 | 0.34 |
| Bulgaria | Upper middle income | 8860 | 0.54 | 0.55 | 0.47 | 0.52 | 0.33 |
| Sudan | Lower middle income | 1560 | 0.54 | 0.42 | 0.26 | 0.39 | 0.33 |
| Bosnia and Herzegovina | Upper middle income | 5740 | 0.53 | 0.51 | 0.39 | 0.47 | 0.33 |
| Senegal | Lower middle income | 1410 | 0.62 | 0.36 | 0.19 | 0.35 | 0.32 |
| Ghana | Lower middle income | 2130 | 0.56 | 0.21 | 0.32 | 0.33 | 0.32 |
| Trinidad and Tobago | High income: non-OECD | 15950 | 0.61 | 0.38 | 0.26 | 0.39 | 0.32 |
| Guyana | Upper middle income | 4770 | 0.58 | 0.34 | 0.26 | 0.37 | 0.32 |
| Uganda | Low income | 620 | 0.54 | 0.26 | 0.30 | 0.34 | 0.32 |
| Gambia, The | Low income | 710 | 0.68 | 0.32 | 0.22 | 0.36 | 0.32 |
| Comoros | Lower middle income | 1380 | 0.53 | 0.27 | 0.32 | 0.36 | 0.32 |
| Tajikistan | Low income | 1010 | 0.73 | 0.29 | 0.20 | 0.35 | 0.32 |
| Malawi | Low income | 360 | 0.51 | 0.26 | 0.32 | 0.35 | 0.31 |
| Burkina Faso | Low income | 670 | 0.51 | 0.28 | 0.29 | 0.35 | 0.31 |
| Ukraine | Lower middle income | 2660 | 0.40 | 0.47 | 0.39 | 0.42 | 0.31 |
| Russian Federation | Upper middle income | 10230 | 0.60 | 0.37 | 0.38 | 0.43 | 0.31 |
| Algeria | Upper middle income | . | 0.59 | 0.26 | 0.27 | 0.35 | 0.31 |
| Benin | Low income | 870 | 0.49 | 0.19 | 0.35 | 0.32 | 0.31 |
| Albania | Upper middle income | 4860 | 0.53 | 0.40 | 0.31 | 0.40 | 0.31 |
| Nigeria | Lower middle income | 1960 | 0.45 | 0.25 | 0.28 | 0.31 | 0.30 |
| Madagascar | Low income | 510 | 0.40 | 0.41 | 0.26 | 0.35 | 0.30 |
| Cameroon | Lower middle income | 1440 | 0.46 | 0.21 | 0.33 | 0.32 | 0.29 |
| Guatemala | Upper middle income | 4400 | 0.54 | 0.39 | 0.23 | 0.36 | 0.29 |
| Serbia | Upper middle income | 6390 | 0.59 | 0.42 | 0.39 | 0.46 | 0.29 |
| Georgia | Upper middle income | 4440 | 0.75 | 0.33 | 0.25 | 0.40 | 0.29 |
| Nepal | Low income | 970 | 0.52 | 0.31 | 0.28 | 0.36 | 0.29 |
| Chad | Low income | 670 | 0.42 | 0.26 | 0.27 | 0.31 | 0.29 |
| Papua New Guinea | Lower middle income | 2570 | 0.51 | 0.46 | 0.17 | 0.34 | 0.29 |
| Belarus | Upper middle income | 5670 | 0.67 | 0.27 | 0.32 | 0.38 | 0.29 |
| Bangladesh | Lower middle income | 1750 | 0.37 | 0.36 | 0.31 | 0.35 | 0.29 |
| Maldives | Upper middle income | 9280 | 0.58 | 0.24 | 0.32 | 0.35 | 0.28 |
| Moldova | Lower middle income | 2980 | 0.53 | 0.32 | 0.20 | 0.33 | 0.28 |
| Cuba | Upper middle income | . | 0.58 | 0.43 | 0.17 | 0.35 | 0.28 |
| Armenia | Upper middle income | 4230 | 0.70 | 0.23 | 0.33 | 0.37 | 0.28 |
| Lesotho | Lower middle income | 1390 | 0.60 | 0.39 | 0.20 | 0.36 | 0.28 |
| Tunisia | Lower middle income | 3500 | 0.58 | 0.23 | 0.28 | 0.34 | 0.28 |
| Mali | Low income | 840 | 0.40 | 0.23 | 0.32 | 0.31 | 0.28 |
| Guinea | Low income | 850 | 0.53 | 0.26 | 0.21 | 0.30 | 0.28 |
| Syrian Arab Republic | Low income | . | 0.51 | 0.24 | 0.22 | 0.30 | 0.27 |
| Kyrgyz Republic | Lower middle income | 1220 | 0.48 | 0.28 | 0.33 | 0.36 | 0.27 |
| Zambia | Lower middle income | 1430 | 0.59 | 0.21 | 0.22 | 0.30 | 0.27 |
| Paraguay | Upper middle income | 5670 | 0.51 | 0.14 | 0.37 | 0.30 | 0.27 |
| Myanmar | Lower middle income | 1310 | 0.57 | 0.30 | 0.19 | 0.32 | 0.27 |
| Venezuela, RB | Upper middle income | . | 0.40 | 0.52 | 0.16 | 0.33 | 0.26 |
| Lao PDR | Lower middle income | 2450 | 0.54 | 0.18 | 0.38 | 0.33 | 0.26 |
| Mongolia | Lower middle income | 3660 | 0.59 | 0.30 | 0.19 | 0.32 | 0.26 |
| Pakistan | Lower middle income | 1590 | 0.44 | 0.25 | 0.24 | 0.30 | 0.25 |
| Congo, Dem. Rep. | Low income | 490 | 0.49 | 0.17 | 0.27 | 0.28 | 0.25 |
| Central African Republic | Low income | 490 | 0.52 | 0.27 | 0.15 | 0.27 | 0.25 |
| Somalia | Low income | . | 0.51 | 0.24 | 0.16 | 0.27 | 0.25 |
| Zimbabwe | Lower middle income | 1790 | 0.50 | 0.27 | 0.15 | 0.28 | 0.24 |
| Bolivia | Lower middle income | 3370 | 0.52 | 0.22 | 0.21 | 0.29 | 0.24 |
| Haiti | Low income | 800 | 0.47 | 0.26 | 0.15 | 0.26 | 0.24 |
| Lebanon | Upper middle income | 7920 | 0.50 | 0.20 | 0.35 | 0.33 | 0.24 |
| Guinea-Bissau | Low income | 750 | 0.49 | 0.18 | 0.21 | 0.26 | 0.24 |
| Burundi | Low income | 280 | 0.44 | 0.31 | 0.13 | 0.26 | 0.24 |
| Solomon Islands | Lower middle income | 2020 | 0.43 | 0.28 | 0.33 | 0.34 | 0.23 |
| Gabon | Upper middle income | 6830 | 0.53 | 0.30 | 0.15 | 0.28 | 0.23 |
| Congo, Rep. | Lower middle income | 1640 | 0.39 | 0.21 | 0.22 | 0.26 | 0.23 |
| Niger | Low income | 390 | 0.51 | 0.20 | 0.14 | 0.24 | 0.22 |
| Equatorial Guinea | Upper middle income | 6840 | 0.58 | 0.25 | 0.15 | 0.28 | 0.22 |
| Liberia | Low income | 610 | 0.40 | 0.24 | 0.13 | 0.23 | 0.21 |
| Yemen, Rep. | Low income | . | 0.22 | 0.21 | 0.22 | 0.22 | 0.19 |
| Mauritania | Lower middle income | 1160 | 0.41 | 0.11 | 0.23 | 0.22 | 0.19 |
| Cambodia | Lower middle income | 1390 | 0.31 | 0.12 | 0.27 | 0.21 | 0.18 |
| Sierra Leone | Low income | 490 | 0.41 | 0.17 | 0.12 | 0.20 | 0.17 |
| Angola | Lower middle income | 3370 | 0.39 | 0.25 | 0.07 | 0.19 | 0.17 |
| Iraq | Upper middle income | 5040 | 0.43 | 0.10 | 0.12 | 0.17 | 0.15 |
| Libya | Upper middle income | 6400 | 0.58 | 0.09 | 0.12 | 0.18 | 0.15 |
| Afghanistan | Low income | 550 | 0.44 | 0.05 | 0.05 | 0.10 | 0.09 |
